# Analyse des Einflusses der zunehmenden Feminisierung im Gesundheitswesen auf die Urologie

**DOI:** 10.1007/s00120-022-01931-3

**Published:** 2022-09-09

**Authors:** M. Himmler, D. Schultz-Lampel, E. Hellmis, K. F. Kowalewski, M. S. Michel, S. Weinberger

**Affiliations:** 1 Klinik für Urologie und Urochirurgie, Universitätsmedizin Mannheim, Universität Heidelberg, Theodor-Kutzer-Ufer 1–3, 68167 Mannheim, Deutschland; 2 Kontinenzzentrum Südwest, Schwarzwald-Baar Klinikum, Villingen-Schwenningen, Deutschland; 3 Urologicum Duisburg, Fahrner Str. 123, Duisburg, Deutschland; 4 Klinik für Urologie, Campus Benjamin Franklin, Charité Universitätsmedizin Berlin, Berlin, Deutschland

**Keywords:** Genderaspekte, Genderratio, Zufriedenheit, Chancengleichheit, Arbeitszeitmodell, Gender aspects, Gender ratio, Satisfaction, Equal opportunities, Working time model

## Abstract

**Hintergrund und Fragestellung:**

Ziel dieser wissenschaftlichen Arbeit war es, Genderaspekte und Trends in Klinik, Forschung und Niederlassung in der Urologie zu analysieren. Dabei lag der Fokus auf der Objektivierung des genderspezifischen Wandels im Fachgebiet „Urologie“ zum aktuellen Zeitpunkt und in der Zukunft.

**Material und Methoden:**

Es erfolgte eine digitale Umfrage bei urologischen Ärzt:innen in Deutschland über das Portal SurveyMonkey©, welche über den E‑Mail-Verteiler der Deutschen Gesellschaft für Urologie e. V. (DGU) und des Berufsverbands der Deutschen Urologen e. V. (BvDU) an alle eingetragenen Mitglieder verschickt wurde. Es wurden Basisdaten im ambulanten und stationären Sektor erhoben, sowie geschlechtsspezifische Daten in Bezug auf Arbeitsplatzverteilung, Ziele, Zufriedenheit und Gründe für berufliche Entscheidungen.

**Ergebnisse:**

Die Auswertung von 398 Antworten ergab, dass urologische Kolleg:innen in der Niederlassung seltener weiblich (23,6 %) und deutlich älter (mittleres Alter 53 Jahre) waren als im stationären Sektor (Frauenanteil 47,2 %, mittleres Alter 43 Jahre). Niedergelassene Vertragsärzt:innen waren mehr Männer (49,4 %) als Frauen (29,9 %) und die Niederlassung wurde von mehr Männern als Berufswunsch angegeben (28,1 % vs. 22,8 %). Die Gründe für die Niederlassung lagen bei Frauen häufiger im familiären Bereich als bei den Männern (Hauptgründe gute Gelegenheit oder Berufswunsch). Frauen arbeiteten häufiger Teilzeit (27,0 % vs. 11,5 %) und strebten häufiger eine Karriere als Oberärztin an (29,1 % der Frauen, 9,4 % der Männer). Entsprechend war der Wunsch nach einer Habilitation oder Professur bei den Frauen häufiger als bei den Männern (20,5 % vs. 15 %). Signifikant mehr Urologinnen sahen eine Ungleichheit bei den beruflichen Aufstiegschancen (59,7 % vs. 17,5 %, *p* < 0,001) und 73,3 % (vs. 18,5 % der Männer, *p* < 0,001) empfanden ihr Geschlecht als Ursache einer Benachteiligung. Dies führte zu einer signifikant geringeren Zufriedenheit von Frauen mit ihrem beruflichen Status (*p* = 0,008), sowie einem geringeren Gefühl der Wertschätzung (*p* < 0,001).

**Schlussfolgerung:**

Um die Urologie zukunftsfähig zu machen ist es essenziell, Genderaspekte noch stärker zu berücksichtigen. Der eingeschlagene Weg, der nächsten Generation von Urolog:innen ein modernes Fachgebiet zu bieten, in dem alle Ärzt:innen unabhängig von ihrem Geschlecht gerne arbeiten, wertgeschätzt werden und Chancengleichheit herrscht, sollte unbedingt weiter verfolgt und intensiviert werden, um die Urologie für die Zukunft gut aufzustellen.

**Zusatzmaterial online:**

Die Online-Version dieses Beitrags (10.1007/s00120-022-01931-3) enthält eine Tabellarische Darstellung der vollständigen Umfrageergebnisse „Genderaspekte in Klinik und Niederlassung“.

## Abstract (en)

**Background and objective:**

The aim of this study was to evaluate gender aspects and trends in urological clinics, research and private practice. The focus was on the objectification of gender-specific changes in the field of urology at the current time and in the future.

**Material and methods:**

A digital survey among urological physicians in Germany was conducted with the SurveyMonkey© portal, which was sent to all registered members via the email distribution lists of the German Society of Urology (DGU) and the Professional Association of German Urologists (BvDU). Baseline data were collected in the outpatient and inpatient sectors, as were gender-specific data related to job distribution, goals, satisfaction, and reasons for career decisions.

**Results:**

The analysis of 398 responses revealed that urological colleagues in private practice were less likely to be female (23.6%) and significantly older (mean age 53 years) than in the inpatient sector (female proportion 47.2%, mean age 43 years). More men (49.4%) than women (29.9%) were in private practice and this was indicated as a career aspiration by more men than women (28.1% vs. 22.8%). The reasons for setting up a practice were more often family-related for women than for men (main reasons good opportunity or career aspiration). Women were more likely to work part-time (27.0% vs. 11.5%) and more likely to aspire to a career as a senior physician (29.1% of women, 9.4% of men). Correspondingly, the desire for a postdoctoral position or professorship was more common among women than among men (20.5% vs. 15%). Significantly more female urologists perceived inequality in career advancement opportunities (59.7% vs. 17.5%, *p* < 0.001) and 73.3% (vs. 18.5% of men, *p* < 0.001) perceived their gender as a cause of disadvantage. This resulted in significantly lower satisfaction of women with their professional status (*p* = 0.008) as well as a lower feeling of being valued (*p* < 0.001).

**Conclusion:**

In order to prepare our specialty field for the future, it is essential to give even greater consideration to gender aspects. The path taken to offer the next generation of urologists a modern specialty in which all physicians, regardless of their gender, enjoy working, are valued and where equal opportunities prevail, should definitely be pursued and intensified in order to position urology well for the future.

## Einleitung

Seit Jahrzehnten zeichnet sich ein deutlicher Trend in der Humanmedizin ab: sie wird weiblich [1]. Im Wintersemester 2020/2021 lag der Frauenanteil der Medizinstudierenden in Deutschland bei knapp 64 % [2], in den USA überstieg der Frauenanteil bei den Medizinstudent:innen gemäß der Association of American Medical Colleges (AAMC) erstmals 2019 die 50 %-Marke [3]. Während einige medizinische Fächer seit jeher einen hohen Frauenanteil aufweisen (Deutschland Jahr 2021 Gynäkologie 70,9 %, Pädiatrie 62,2 %, Dermatologie 58,9 % [4]), liegt dieser insbesondere in vielen chirurgischen Fächern immer noch deutlich niedriger (Jahr 2021 Orthopädie und Unfallchirurgie 19,1 %, Neurochirurgie 20,9 % [4]). Als Gründe dafür werden unter anderem männliche Dominanz im Fachgebiet, Schwierigkeiten in der Vereinbarkeit von Familie und Beruf, begrenzte flexible Ausbildungsmöglichkeiten und wenig weibliche Vorbilder verantwortlich gemacht [5]. Der Frauenanteil in der Urologie ist in den letzten Jahren erfreulicherweise angestiegen. Waren 2014 noch 15,1 % Ärztinnen in der Urologie tätig, so waren es 2018 bereits 18,2 % und 2021 20,6 % [4]. Insgesamt liegt der Anteil prozentual dennoch weiterhin verhältnismäßig niedrig [4, 6], obwohl auch bei den abgelegten Facharztprüfungen für Urologie der Trend deutlich ist: Während 2014 noch 29,6 % der deutschen urologischen Facharztprüflinge weiblich waren, erhöhte sich dies auf 32,5 % im Jahr 2017 und sogar 38,3 % im Jahr 2019 [4].

Im Jahr 1911 wurde mit Dora Brücke-Teleky die erste weibliche Urologin als Mitglied in die Deutsche Gesellschaft für Urologie e. V. (DGU) aufgenommen [7]. Seitdem hat sich durch den stetig wachsenden Anteil an Frauen vieles in der Urologie in Deutschland verändert. Die Genderthematik ist in den letzten Jahren auch in der Urologie immer weiter in den Fokus gerückt. Dies führte u. a. dazu, dass die Fachzeitschrift *Der Urologe* nun in *Die Urologie* umbenannt wurde [8]. Weiterhin soll es auf dem kommenden deutschen Urologiekongress 2022 nach Möglichkeit erstmals keine rein männlich besetzten Panels bei den Vorsitzenden (sog. „Manels“) mehr geben, wie es auch auf dem Europäischen Urologiekongress (EAU) schon länger üblich ist.

Um den genderspezifischen Wandel des Fachgebiets „Urologie“ in der Zukunft besser quantifizieren zu können, ist es Ziel dieser Studie, Genderaspekte und Trends in Klinik, Forschung, Niederlassung und urologischen Randbereichen zum aktuellen Zeitpunkt zu erfassen. Hierbei soll der Schwerpunkt insbesondere auf genderspezifischen Unterschieden im beruflichen Status, Arbeitszeitmodellen, Spezialisierung, Aufstiegschancen und speziellen Aspekten der Niederlassung liegen. Eine derartige systematische Erhebung für die Urologie hat nach Wissen der Autoren in Deutschland bisher nicht stattgefunden.

## Material und Methoden

### Studienpopulation

Zielgruppe der digitalen Umfrage waren alle urologischen Ärzt:innen in Deutschland. Durch die breite Befragung sollte die Erfassung von Genderaspekten sowohl in der Klinik als auch in der Forschung, Niederlassung und in Randbereichen gewährleistet sein. Die Abfrage erfolgte über das Portal SurveyMonkey© (Survey Monkey Inc., San Mateo, CA, USA). Der Aufruf zur Teilnahme erfolgte über den Newsletter der DGU, sowie über den Newsletter des Berufsverbands der Deutschen Urologen e. V. (BvDU) an alle eingetragenen Mitglieder. Über die DGU wurden 5072 Mitglieder angeschrieben, über den BvDU 2498 Mitglieder. Die Umfrage war über einen Link erreichbar und über die IP-Adresse auf eine einmalige Teilnahme beschränkt. Sie erfolgte komplett anonym. Ab dem 31.05.2022 war die Umfrage für 14 Tage geöffnet.

### Fragebogen

Die Befragung umfasste insgesamt 47 Fragen, wobei 32 Fragen für alle Teilnehmer geeignet waren und 15 Fragen nur für Kolleg:innen in der Niederlassung konzipiert wurden. Es wurden 27 Multiple-choice-Fragen, 17 Fragen mit Option einer Mehrfachnennung und 3 offene Fragen gestellt.

Es wurden folgende Bereiche abgefragt:

1. Basisfragen (3 Fragen: Geschlecht, Alter, Bundesland),
2. beruflicher Status (10 Fragen: beruflicher Status, Zufriedenheit mit dem beruflichen Status, angestrebter beruflicher Status, aktueller Sektor, Zufriedenheit mit dem Sektor, Grund für Entscheidung zu diesem Sektor, akademischer Grad, Zufriedenheit mit dem akademischen Grad, angestrebter akademischer Grad, Gremienarbeit),
3. Arbeitszeitmodelle (4 Fragen: aktuelles Arbeitszeitmodell, gewünschtes Arbeitszeitmodell, Zufriedenheit mit dem Arbeitszeitmodell, Grund für die Entscheidung zu diesem Arbeitszeitmodell),
4. Spezialisierung (3 Fragen: aktuelle Spezialisierung, Grund für die Entscheidung zu dieser Spezialisierung, geschlechterspezifische Unterschiede in der Spezialisierung),
5. Genderverteilung (3 Fragen: Genderratio, angestrebte Genderratio, Entwicklung Frauenanteil),
6. Arbeitsklima (2 Fragen: aktuelles Arbeitsklima, Entwicklung Arbeitsklima über 5 Jahre),
7. Genderaspekte (7 Fragen: Wahl Urologie bei Frauen, Wahl Urologie bei Männern, Aufstiegschancen, Leistung, Wertschätzung, Beförderungsunterschiede, Gründe dafür),
8. Niederlassung (15 Fragen: Grund für die Niederlassung, beruflicher Status zum Zeitpunkt des Wechsels, momentane Stellung, Hybridmodelle, flexible Arbeitszeiten, Konsiliardienst, Belegmodelle, gleichberechtigtes Arbeiten, Patientenzuteilung, Zulassungen, Spezialsprechstunden, operative Tätigkeit, Arbeit mit Lebenspartner:in, anteilige Vergütung, Einkommensunterschiede).

### Statistik

Die Ergebnisse wurden mit Microsoft Excel® (Microsoft Corporation, Redmond, WA, USA) und SurveyMonkey© aufbereitet. In der Auswertung wurden bei Mehrfachantwortmöglichkeiten stets alle Antworten berücksichtigt. Es wurde eine deskriptive Statistik durchgeführt. Hierbei wurden quantitative Variablen als Mittelwert und Standardabweichung (SD) bzw. Median und „interquartile range“ (IQR) angegeben, kategoriale Variablen als absolute und relative Häufigkeiten. Bei Mehrfachantwortmöglichkeiten beziehen sich in allen Tabellen die relativen Häufigkeiten auf die Anzahl der Antworten, nicht auf die Anzahl der Befragten. Bei Gruppenvergleichen wurde der t‑Test für unverbundene Stichproben für kontinuierliche Variablen und der χ^2^-Test für kategorische Variablen angewendet. Alle statistischen Analysen wurden durch die Software *R* (Version 4.2.0, R Foundation for Statistical Computing, Vienna, Austria) durchgeführt [9]. Das Signifikanzniveau wurde auf *p* ≤ 0,05 festgelegt.

## Ergebnisse

### Demografische Daten in Niederlassung und Klinik

Insgesamt gingen 398 Antworten von 127 Frauen und 267 Männern ein. 4 Urolog:innen machten keine Angabe zu ihrem Geschlecht. 253 Urolog:innen gaben an, im ambulanten Bereich tätig zu sein, während 126 Urolog:innen im stationären Sektor arbeiteten (Tab. 1). 19 Urolog:innen gaben anderweitige Tätigkeitsbereiche an. Das mittlere Alter der im ambulanten Sektor arbeitenden Kolleg:innen war mit 53,0 ± 10,0 (Range 27–79) Jahren um 10 Jahre höher als das der in der Klinik arbeitenden Urolog:innen (42,9 ± 11,8; Range 26–82 Jahre). Im ambulanten Bereich betrug der Frauenanteil 23,6 %, während er unter den Klinikangestellten bei 47,2 % und somit doppelt so hoch lag. In der Wahrnehmung der befragten niedergelassenen Kolleg:innen ist der Frauenanteil in den letzten 5 Jahren tendenziell eher gleich geblieben (49,2 %), während er im stationären Bereich eher als deutlich oder leicht steigend wahrgenommen wird (25,4 % und 30,2 %).

**Table 1:** 

	Niederlassung (*n* = 253)	Klinik (*n* = 126)
**Variable**	**Mean (SD)**	**Mean (SD)**
*Alter (n* *=* *397)*	52,97 (9,98)	42,90 (11,77)
*Ärzt:innen an der Arbeitsstätte (n* *=* *385)*		
– Weiblich	1,71 (2,39)	6,08 (6,46)
– Männlich	3,64 (4,21)	11,23 (7,69)
– Divers	0,01 (0,09)	0,17 (0,92)
**Variable**	**n (%)**	**n (%)**
*Geschlecht (n* *=* *395)*		
– Männlich	191 (76,4)	66 (52,8)
– Weiblich	59 (23,6)	59 (47,2)
– Divers	0 (0,0)	0 (0,0)
*Veränderung des Frauenanteils an der Arbeitsstätte über 5 Jahre (n* *=* *398)*		
– Deutlich gestiegen	53 (21,0)	32 (25,4)
– Leicht gestiegen	40 (15,9)	38 (30,2)
– Gleichgeblieben	124 (49,2)	34 (27,0)
– Gefallen	12 (4,8)	15 (11,9)
– Kann ich nicht beurteilen	23 (9,1)	7 (5,6)

Mögliche Abweichungen in Summenscores kommen durch fehlende Daten zustande

*n* Anzahl, *Mean* Mittelwert, *SD* Standardabweichung

### Genderaspekte in Klinik und Niederlassung

In Tab. 1 sind die statistisch signifikanten geschlechterspezifischen Unterschiede der Befragung dargestellt. In der vorliegenden Umfrage gaben Frauen auf die Frage nach ihrem aktuellen beruflichen Status seltener als ihre männlichen Kollegen einen Oberarztstatus (15,7 % vs. 17,2 %) oder einen Chefarztstatus (1,6 % vs. 13,5 %) an. 49,4 % der Männer gaben an, Praxisinhaber oder Praxisteilhaber zu sein, bei den Frauen lag der Prozentsatz mit 29,9 % (38/127) deutlich niedriger. Die Frage nach dem angestrebten beruflichen Status wurde von 25/267 (9,4 %) der Männer mit „Oberarzt“ beantwortet, während 37/127 (29,1 %) der Frauen eine Position als Oberärztin anstreben. 21/267 (7,9 %) Männer und 7/127 (5,5 %) Frauen streben eine Position als Chef:ärztin an. Die Selbstständigkeit als Praxisinhaber:in oder -teilhaber:in wurde von 75/267 (28,1 %) Männern und 29/127 (22,8 %) Frauen als angestrebter beruflicher Status angegeben. Männer präferieren in diesem Kontext eher eine Praxisinhaberschaft (52/75) als eine Teilhaberschaft (23/75), wohingegen Frauen fast gleichermaßen eine Inhaber- oder Teilhaberschaft präferieren (14/29 und 15/29). Auf die Frage, warum sie in ihrem aktuellen Sektor tätig sind, war im Gesamtkollektiv „Gelegenheit“ die häufigste Antwort. Bei den Männern lagen „Gelegenheit“ und „Berufswunsch“ als häufigste Gründe für die Niederlassung mit je 19,6 % gleichauf, bei Frauen war die häufigste Antwort „familiäre Gründe“ (17,6 %), gefolgt von „geringere Arbeitsbelastung/keine Dienste“ (16,2 %).

Betrachtet man den höchsten akademischen Grad der Befragten, so sind 55/127 (43,3 %) der Frauen und 95/267 (35,6 %) der Männer promoviert, 39/267 (14,6 %) der Männer und 9/127 (7,1 %) der Frauen habilitiert oder Inhaber:in einer Professur. Auf die Frage, welchen akademischen Grad sie zukünftig anstreben, antworteten 40/267 (15,0 %) Männern und 26/127 (20,5 %) Frauen mit „Habilitation“ oder „Professur“. 34/126 (27,0 %) der teilnehmenden Frauen arbeiten aktuell in Teilzeit, während mit 55/124 (44,3 %) deutlich mehr Frauen angaben, dass ihr präferiertes Arbeitszeitmodell „in Teilzeit“ wäre. Bei den Männern arbeiten 11,5 % der Befragten aktuell in Teilzeit, gewünscht wäre es von 32,3 %. Der Grund für die Wahl des aktuellen Arbeitszeitmodells war bei Männern mit 43,2 % (131/267) am häufigsten der, dass es ihrer Wunschvorstellung entspricht. Auch bei Frauen war „Wunschvorstellung“ die häufigste Antwort (29,7 %), knapp gefolgt von „besseren Vereinbarkeit mit dem Privatleben“ (27,2 %) bzw. „Vorgabe des Arbeitgebers“ (22,8 %).

Mögliche Aufstiegschancen wurden bei Frauen und Männern signifikant unterschiedlich wahrgenommen. Während 82,5 % der Männer gleiche Aufstiegschancen für ihre weiblichen Kollegen sehen, wird dies nur von 40,3 % der Frauen so wahrgenommen. Männer waren zu 84,6 % der Meinung, dass sie und ihre weiblichen Kollegen die gleiche Leistung erbringen, Frauen waren zu 43,9 % der Meinung, dass sie mehr leisten als ihre männlichen Kollegen. Mit 73,3 % gaben signifikant mehr Frauen an, dass ihrer Meinung nach ihr Geschlecht einen Einfluss bei einer ausbleibenden Beförderung hat, während 81,5 % der Männer annehmen, dass eine Entscheidung zur Beförderung unabhängig vom Geschlecht erfolgt (*p* < 0,001). Als optimales Verhältnis weiblicher zu männlicher Mitarbeiter in einer Arbeitsstätte wird von Männern und Frauen gleichermaßen ein ausgewogenes Geschlechterverhältnis von 50/50 angegeben.

### Zufriedenheit, Arbeitsklima und Wertschätzung

**Figure 1:**
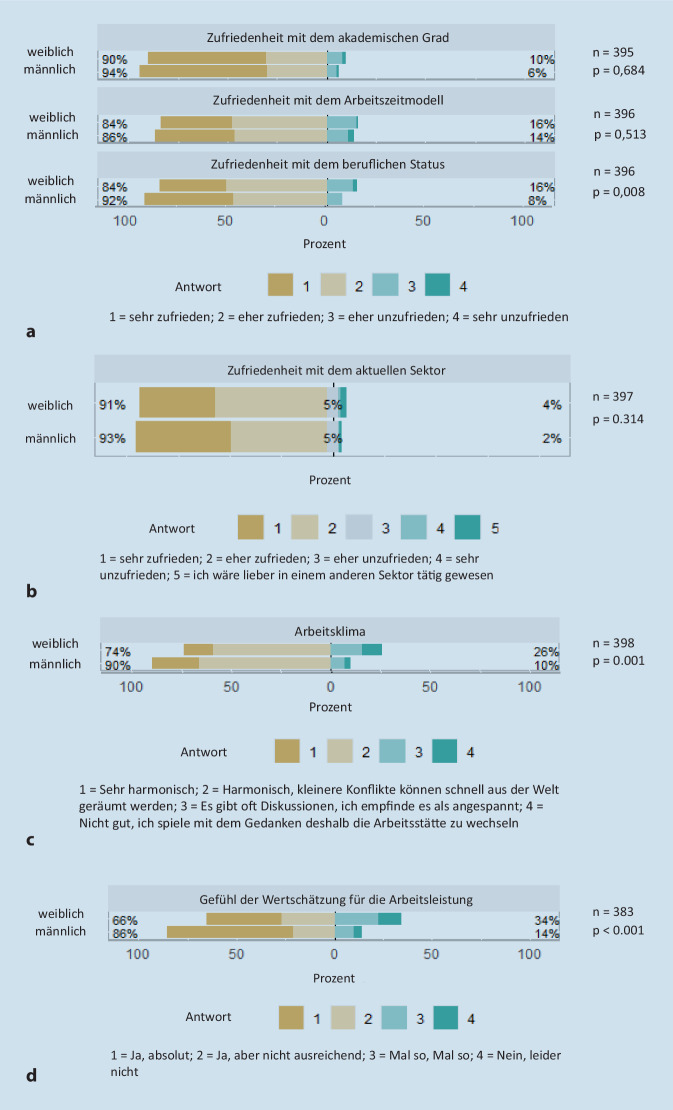

Sowohl bei Urologinnen als auch bei Urologen besteht prinzipiell eine sehr hohe Zufriedenheit im Beruf. „Sehr zufrieden“ oder „eher zufrieden“ mit ihrem akademischen Grad sind 90 % der Frauen und 94 % der Männer (*p* = 0,684; Abb. 1a). Mit ihrem Arbeitszeitmodell sind 84 % der Frauen und 86 % der Männer zufrieden (*p* = 0,513). 91 % der Frauen und 93 % der Männer sind mit ihrem aktuellen Sektor zufrieden (*p* = 0,314; Abb. 1b). Dahingegen unterschied sich die Zufriedenheit mit dem beruflichen Status bei Männern und Frauen signifikant (92 % vs. 84 %; *p* = 0,008; Abb. 1a). Die Entwicklung des Arbeitsklima in den letzten 5 Jahre wird von Frauen signifikant weniger gut wahrgenommen als von ihren männlichen Kollegen (74 % vs. 90 %; *p* = 0,001; Abb. 1c). Außerdem fühlen sich Frauen für Ihre Arbeitsleistung signifikant weniger wertgeschätzt, als das bei Männern der Fall war (66 % vs. 86 %, *p* < 0,001; Abb. 1d).

## Diskussion

Die Befragung zeigt klare Ergebnisse, die sich durch aktuelle Daten in der Literatur untermauern lassen.

### Männer sind häufiger als Vertragsärzte niedergelassen und Frauen häufiger in der Klinik angestellt

Unter den Teilnehmer:innen unserer Befragung arbeiteten deutlich mehr Frauen im stationären als im ambulanten Bereich. Der Frauenanteil unter den niedergelassenen Vertragsärzten war nochmal geringer. Auf die Frage nach dem zukünftig angestrebten beruflichen Status antworteten 23,5 % der Männer und nur 12,3 % der Frauen mit „Praxisinhaber:in“, Männer hatten also doppelt so häufig als Frauen den Wunsch nach einer eigenen Praxis. Dieser Trend wird durch die Daten der Ärztestatistik der Bundesärztekammer (BÄK) untermauert. So gab es 2021 in Deutschland insgesamt 6467 Urolog:innen. Davon war etwas mehr als die Hälfte (51,9 %) ambulant tätig und wiederum die überwiegende Mehrheit davon als Praxisinhaber:in oder -teilhaber:in niedergelassen [4]. 43,1 % Urolog:innen waren in der stationären Versorgung tätig. Insgesamt gab es im Jahr 2021 1335 Urologinnen in Deutschland (Frauenanteil 20,6 %), von denen wie auch in unserer Studie deutlich weniger ambulant als stationär tätig waren (*n* = 544, 40,7 % vs. *n* = 683, 51,2 %; [4]). Zudem waren Frauen deutlich seltener niedergelassen als dies bei den männlichen Kollegen der Fall war (58,1 % vs. 86,3 %); insgesamt lag 2021 die Frauenquote bei 11,5 % unter den Vertragsärzt:innen in der Niederlassung. Im Gegensatz dazu waren 41,9 % der ambulant tätigen Urologinnen angestellt; unter den Männern lag der Anteil der ambulant angestellten Ärzte nur bei 13,7 % [4]. Diese Zahlen zeigen, dass die im Rahmen der Ärztinnenstatisik erhobenen Daten, welche die Existenzgründungen von Praxen evaluierten, offenbar nicht generalisiert auf alle Fächer übertragbar sind: Die Studie hatte ergeben, dass 65,5 % der Existenzgründungen von Frauen Einzelpraxen waren und 34,6 % in Kooperation erfolgten [10]; eine Situation, die sich in der Urologie nicht widerspiegelt.

Sucht man nach dem Grund für die eigene Niederlassung, so war dieser bei Männern eher ihrem persönlichen Wunsch entsprechend: Die Antworten „Gelegenheit“ und „Berufswunsch“ wurden von den Männern in Niederlassung am häufigsten angegeben. Frauen hingegen entschieden sich häufig aufgrund äußerer Umstände wie der „familiären Situation“ und der „Arbeitsbelastung“ für die Niederlassung.

Ein ähnliches Bild zeigte sich beim Arbeitszeitmodell: der höhere Anteil von Frauen in Teilzeit (27,0 % vs. Männer 11,5 %) verwundert nicht. Auf die Frage nach dem gewünschten Arbeitszeitmodell antworteten 32,3 % der Männer und 55,6 % der Frauen mit „Teilzeit“. Der Anteil der Frauen, die in Teilzeit arbeiten wollen, lag schon im Absolventenreport Medizin vor knapp 30 Jahren bei ca. 50 % und hat sich somit seither nicht wirklich verändert [11]. Neu ist, dass damals nur 19 % der Männer [11], in der vorliegenden Studie aber 32,2 % einen Teilzeitwunsch äußerten. Dies spiegelt einmal mehr den Generationenkonflikt wider: Bei den Generationen Y und Z findet ein Umdenken mit dem Wunsch weg von kompletter Aufopferung im Beruf hin zu besserer Vereinbarkeit von Beruf und Privatleben statt. Dies stößt bei der älteren Generation häufig auf Unverständnis und könnte als Desinteresse oder Arbeitsunlust fehlgedeutet werden. Die aktive Umsetzung von Teilzeitmodellen auch für Männer setzt sich zumindest im stationären Sektor noch nicht flächendeckend durch und die Äußerung nach einem Teilzeitwunsch vor dem/der Vorgesetzten dürfte vielen Männern aus Sorge vor Unverständnis oder dadurch bedingte schlechteren Karrierechancen weiterhin schwerfallen.

### Genderunterschiede im stationären Sektor

Betrachtet man den höchsten akademischen Grad der Befragten, so sind 14,6 % der Männer und 7,1 % der Frauen in der aktuellen Umfrage habilitiert und/oder Inhaber:in einer Professur, angestrebt wird dies von 15,0 % der Männer und 20,5 % der Frauen. Eine Untersuchung des statistischen Bundesamtes ergab, dass im Jahr 2020 32 % aller Habilitationen in der Humanmedizin von Frauen absolviert wurden, was zunächst erfreulich ist [2]. Jedoch zeigte eine Studie, dass in Deutschland Frauen deutlich häufiger in konservativen Fächern wie Pädiatrie oder Augenheilkunde habilitieren, als dies in chirurgischen Fächern der Fall ist [12]. Gemäß der DGU-Datenbank (Stand 29.07.2021) wurden in Deutschland zwischen 2013 und 2020 insgesamt 138 Habilitationsverfahren in der Urologie abgeschlossen, 14,5 % davon von Frauen. Hinzu kommt, dass Publikationen von Urologinnen signifikant seltener in hochrangigen Journals publiziert werden, wie eine aktuelle Studie ergab [13].

Obwohl Frauen prozentual gesehen häufiger als ihre männlichen Kollegen im stationären Bereich arbeiten, war unter den befragten Urolog:innen der Frauenanteil unter den Oberärzt:innen (30,3 %) und Chefärzt:innen (5,3 %) prozentual geringer. Verglichen mit einer Erhebung aus 2016 an den deutschen Universitätsklinika, die einen Frauenanteil von 15 % unter den urologischen Oberärzt:innen ermittelte [14], womit die Urologie damals das Schlusslicht unter allen Fächern darstellte, zeigt sich hier eine positive Entwicklung, da sich der Anteil in den letzten 6 Jahren fast verdoppelt hat. Dennoch gibt es weiterhin Potenzial zur Verbesserung.

### Der Wunsch nach einer Führungsposition wurde häufiger von Frauen geäußert

In unserer Umfrage gaben 29,1 % der Frauen, und somit 3‑mal mehr als ihre männlichen Kollegen, an, eine Oberarztposition anzustreben, 5,5 % gaben eine Chefarztposition als Berufswunsch an. Unsere Ergebnisse widersprechen vorangegangenen Studien, die ein Fortbestehen traditioneller Geschlechterrollen proklamierten: Männliche Absolventen streben Führungspositionen in Krankenhausabteilungen an, während weibliche Medizinabsolventen den ambulanten Sektor als Arbeitsplatz bevorzugen [15, 16]. Dass der Wunsch nach Führungspositionen bei den Urologinnen sich noch nicht in der Realität umsetzen ließen, zeigt die Statistik der Bundesärztekammer: Der Frauenanteil unter den stationär leitenden Ärzt:innen in Deutschland 2021 lag in der Gynäkologie bei 26,8 %, in der Dermatologie bei 24,7 %, in der Pädiatrie bei 21,9 %, in der Neurochirurgie bei 7,35 %, in der Orthopädie bei 6,1 % und in der Urologie bei 4,9 % [4]. Ein Vergleich mit den Zahlen von 2017 verdeutlicht, dass sich hier in den letzten Jahren wenig verändert hat: im Jahr 2017 lag der Frauenanteil unter den stationär leitenden Ärzt:innen in der Gynäkologie bei 22,1 %, in der Dermatologie bei 25,0 %, in der Pädiatrie bei 17,9 %, in der Neurochirurgie bei 5,6 %, in der Orthopädie bei 4,9 % und in der Urologie bei 3,3 % [4]. Dieses Phänomen zeigte sich ebenfalls in mehreren amerikanischen Studien und wurde bereits ausgiebig adressiert [17, 18]. Projekte zur Verbesserung des Genderverhältnis in leitenden Positionen und an Universitäten wurden ins Leben gerufen [19], welche bisher jedoch nur punktuelle Erfolge verzeichnen und sich nicht breitflächig durchsetzen konnten [10, 20, 21].

Es zeigt sich in unserer Befragung, dass insbesondere von Frauen starke geschlechterspezifische Unterschiede bei den Aufstiegschancen wahrgenommen werden. Nur 40,3 % der Frauen schätzen ihre Aufstiegschancen genauso wie die ihrer männlichen Kollegen ein. 73,3 % der Frauen sehen ihr Geschlecht als Ursache für geringere Aufstiegsmöglichkeiten an; dies ähnelt den Daten einer kanadischen Studie, in der 56 % der weiblichen Befragten ihr Geschlecht als Hauptursache für Diskriminierung empfanden und 57 % angaben, Hindernisse in ihrer beruflichen Karriere durch ihr Geschlecht erfahren zu haben [22]. Hingegen nahmen 81,5 % der Männer in unserer Studie an, dass das Geschlecht bei beiden Geschlechtern keinen Einfluss auf Beförderung oder Aufstiegschancen hat. Schon in der Vergangenheit wurde proklamiert, dass die Entwicklung und Umsetzung längst überfälliger Abhilfemaßnahmen zur Verbesserung der Chancengleichheit von der höchsten Ebenen der Abteilungen und Institutionen kommen muss [23]. Cochran et al. schlussfolgerten in ihrer Studie von 2013, dass das Ausmaß der wahrgenommenen Geschlechterdiskriminierung unter den jüngeren Frauen in der Chirurgie eine Notwendigkeit zur Veränderung der Kultur in den chirurgischen Abteilungen impliziert, wenn die akademische Chirurgie talentierte Frauen anziehen und halten soll, denen alternative Karrieremöglichkeiten in einem günstigeren Umfeld zur Verfügung stehen [23]. Den Ergebnissen unserer Umfrage nach zu schlussfolgern sind wir davon auch heute noch weit entfernt: Ein Gefühl der Ungerechtigkeit, für gute Leistung weniger Chancen zu bekommen, scheint unter den Urologinnen weiterhin vorhanden zu sein. Es gibt viel Potenzial in der deutschen Urologie und es wäre wünschenswert, dieses durch Chancengleichheit, moderne Arbeitszeitmodelle und geschlechtsunabhängige Aufstiegsmöglichkeiten und Förderprogramm zukünftig voll ausschöpfen zu können. Dies wird nur möglich sein, wenn es gelingt, veraltete Vorstellungen und Konzepte zu modernisieren und Frauen und Männern gleichermaßen den Wunsch nach flexibleren Arbeitszeiten, Teilzeitmodellen und eine bessere Vereinbarkeit von Familie und Beruf zu ermöglichen. Dafür benötigen Klinikdirektor:innen und Chefärzt:innen einen größeren Spielraum zur flexiblen Personalplanung, da der Personal- und Kostendruck häufig alle Reformansätze im Keim erstickt.

Die größte Stärke der vorgestellten Studie ist, dass sie unter Urolog:innen in unterschiedlichen Berufsfeldern und an verschiedenen Arbeitsplätzen durchgeführt wurde. Aufgrund der Methodik einer fragebogenbasierten Studie unterliegen die Ergebnisse jedoch einer möglichen Verzerrung durch die Antwortenden („responder bias“). Weiterhin war die Rücklaufquote eher niedrig, sodass die Erkenntnisse nicht als generalisiert angenommen werden können.

## Schlussfolgerung

Die Urologie unterliegt einer zunehmenden Feminisierung, was dem generellen Trend in der Medizin in Deutschland entspricht. Die Generationen Y und Z wollen ihr Leben nicht mehr rund um die Uhr in der Klinik verbringen und legen mehr Wert auf Teilzeitarbeit und die Vereinbarkeit von Familie und Beruf. Um die Urologie zukunftsfähig zu machen ist es daher essenziell, Genderaspekte zu berücksichtigen und gute berufliche Perspektiven für alle Geschlechter zu ermöglichen. Voraussetzung hierfür ist, ein Arbeitsumfeld zu schaffen, in dem alle gleichermaßen gerne arbeiten. Um auch zukünftig eine suffiziente ambulante Versorgung urologischer Patient:innen gewährleisten zu können und dem steigenden Patientenaufkommen gerecht zu werden, sollten insbesondere für Frauen Anreize für eine Niederlassung geschaffen werden. Im stationären Bereich liegt eine wichtige Aufgabe in der Schaffung von geschlechtsunabhängiger Chancengleichheit. Dies sollte berücksichtigen, dass auch viele Männer den Wunsch nach Arbeit in Teilzeit haben. Wenn es gelingt, dem Nachwuchs die Urologie als modernes Fachgebiet anbieten zu können, in dem alle geschlechtsunabhängig gleichermaßen gerne arbeiten, Wertschätzung und Chancengleichheit erfahren, dann ist die Urologie für die Zukunft gut aufgestellt.

## Supplementary Information




