# Supplementary material for: Analysis of the impact of increasing feminization in the healthcare system on urology
Source: Urologie. 2022 Sep 9;61(10):1083–92. [Article in German] doi: 10.1007/s00120-022-01931-3 (PMC9550724; doi:10.1007/s00120-022-01931-3)
Supplement: Supplementary file 1 [file 120_2022_1931_MOESM1_ESM.docx]

**Tabelle S1 Genderaspekte in Klinik und Niederlassung**

| **Variable** |  | **Gesamt**  **n = 398** | **Männlich**  **n = 267** | **Weiblich**  **n = 127** | **p-Wert** |
| --- | --- | --- | --- | --- | --- |
| Aktueller beruflicher Status  (*Mehrfachnennung möglich)*  (n=399) | Arzt/Ärztin in Weiterbildung  Facharzt/Fachärztin  Oberarzt/ Oberärztin  Chefarzt / Chefärztin  Lehrstuhlinhaber/-in  Praxisinhaber /-in  Praxisteilhaber /-in  Angestellt in Praxis/MVZ  Nicht ärztlich tätig (z.B. Forschung)  Clinician Scientist  Sonstiges | 50 (10.3)  111 (22.8)  66 (13.6)  38 (7.8)  3 (0.6)  129 (26.5)  41 (8.4)  26 (5.3)  7 (1.4)  4 (0.8)  12 (2.5) | 17 (5.1)  71 (21.1)  46 (13.7)  36 (10.7)  2 (0.6)  102 (30.4)  30 (8.9)  17 (5.1)  5 (1.5)  1 (0.3)  9 (2.7) | 33 (21.9)  40 (26.5)  20 (13.2)  2 (1.3)  1 (0.7)  27 (17.9)  11 (7.3)  9 (6.0)  2 (1.3)  3 (2.0)  3 (2.0) | < 0.001 |
| Angestrebter beruflicher Status  (n = 340) | Arzt/Ärztin in Weiterbildung  Facharzt/Fachärztin  Oberarzt/ Oberärztin  Chefarzt / Chefärztin  Lehrstuhlinhaber/-in  Praxisinhaber /-in  Praxisteilhaber /-in  Angestellt in Praxis/MVZ  Nicht ärztlich tätig (z.B. Forschung)  Clinician Scientist  Sonstiges | 1 (0.3)  26 (7.7)  63 (18.6)  28 (8.3)  5 (1.5)  66 (19.5)  39 (11.5)  11 (3.2)  11 (3.2)  0 (0.0)  89 (26.3) | 1 (0.5)  12 (5.4)  25 (11.3)  21 (9.5)  3 (1.4)  52 (23.5)  23 (10.4)  7 (3.2)  8 (3.6)  0 (0.0)  69 (31.2) | 0 (0.0)  13 (11.3)  37 (32.5)  7 (6.1)  2 (1.8)  14 (12.3)  15 (13.2)  4 (3.5)  3 (2.6)  0 (0.0)  19 (16.7) | < 0.001 |
| Aktueller Sektor (*Mehrfachnennung möglich)*  (n = 398) | Ambulant  Stationär  Privatwirtschaft / Industrie  Behörde  Labor / Forschung  Sonstiges | 250 (49.5)  222 (44.0)  3 (0.6)  2 (0.4)  17 (3.4)  11 (2.2) | 191 (54.6)  141 (40.3)  3 (0.9)  0 (0.0)  11 (3.1)  4 (1.1) | 59 (38.1)  81 (52.3)  0 (0.0)  2 (1.3)  6 (3.9)  7 (4.5) | < 0.001 |
| Grund für die Niederlassung  (Mehrfachnennung möglich)  (n = 201) | Gelegenheit  Zwang  Unzufriedenheit  Vertrag ausgelaufen  Berufswunsch  Fehlende Aufstiegsmöglichkeiten  Arbeitsbelastung / Dienste  Mehr Urlaubstage / Freizeit  Familiäre Gründe  Finanzielle Gründe | 102 (18.6)  9 (1.6)  49 (8.9)  20 (3.6)  93 (16.9)  45 (8.2)  73 (13.3)  43 (7.8)  75 (13.7)  40 (7.3) | 81 (19.6)  6 (1.5)  36 (8.7)  14 (3.4)  81 (19.6)  34 (8.2)  51 (12.3)  26 (6.3)  51 (12.3)  33 (8.0) | 21 (15.4)  3 (2.2)  13 (9.6)  6 (4.4)  12 (8.8)  11 (8.1)  22 (16.2)  17 (12.5)  24 (17.6)  7 (5.1) | 0.034 |
| Höchster akademischer Grad  (n = 399) | Facharztstatus  Promotion  Habilitation  Professur  Keines der oben genannten | 175 (44.1)  150 (37.8)  22 (5.5)  26 (6.5)  24 (6.0) | 125 (47.0)  95 (35.7)  15 (5.6)  24 (9.0)  7 (2.6) | 48 (37.8)  55 (43.3)  7 (5.5)  2 (1.6)  15 (11.8) | <0.001 |
| Angestrebter akademischer Grad  (n = 282) | Facharztstatus  Promotion  Habilitation  Professur  Sonstiges | 78 (27.8)  52 (18.5)  35 (12.5)  32 (11.4)  84 (29.9) | 38 (21.3)  33 (18.5)  17 (9.6)  23 (12.9)  67 (37.6) | 39 (38.6)  19 (18.8)  17 (16.8)  9 (8.9)  17 (16.8) | 0.001 |
| Arbeitszeitmodell  (n = 390) | Vollzeit  Teilzeit | 325 (83.3)  65 (16.7) | 230 (88.5)  30 (11.5) | 92 (73.0)  34 (27.0) | <0.001 |
| Gewünschtes Arbeitszeitmodell  (n=375) | Vollzeit  Teilzeit | 227 (59.9)  152 (40.1) | 170 (67.7)  81 (32.3) | 55 (44.3)  69 (55.6) | <0.001 |
| Grund für Entscheidung für das Arbeitszeitmodell  (*Mehrfachnennung möglich)*  (n = 391) | Meine Wunschvorstellung  Finanzielle Zwänge  Bessere Vereinbarkeit mit dem Privatleben  Vorgabe des Arbeitgebers  Sonstiges | 178 (38.6)  77 (16.7)  86 (18.7)  81 (17.6)  39 (8.5) | 131 (43.2)  60 (19.8)  43 (14.2)  45 (14.9)  24 (7.9) | 47 (29.7)  17 (10.8)  43 (27.2)  36 (22.8)  15 (9.5) | <0.001 |
| Gleiche Aufstiegschancen für Ärztinnen  (n = 386) | Ja  Nein | 265 (68.8)  120 (31.2) | 212 (82.5)  45 (17.5) | 50 (40.3)  74 (59.7) | <0.001 |
| Gleiche Leistung von Ärztinnen  (n = 375) | Ja, sie leisten genauso viel  Nein, sie leisten weniger  Nein, sie leisten mehr | 279 (74.6)  31 (8.3)  64 (17.1) | 209 (84.6)  29 (11.7)  9 (3.6) | 68 (55.3)  1 (0.8)  54 (43.9) | <0.001 |
| Beförderung von Kolleg:in bei gleich guter Qualifikation (n = 374) | Ja  Nein | 90 (24.1)  283 (75.9) | 36 (14.3)  216 (85.7) | 54 (46.2)  63 (53.8) | <0.001 |
| Geschlecht als Ursache dafür  (n = 124) | Ja  Nein | 54 (70.0)  70 (56.5) | 12 (18.5)  53 (81.5) | 42 (73.3)  15 (26.3) | <0.001 |
| **Variable** |  | **Median (IQR)**  **Gesamt**  **Range** | **Median (IQR)**  **Männlich**  **Range** | **Median (IQR)**  **Weiblich**  **Range** | **p-Wert** |
| Optimale Verteilung weiblicher und männlicher Kolleg:innen  (n = 385) | Weiblich  Männlich | 50 (36 – 50)  0 – 100  50 (50 – 64)  0 – 100 | 50 (30 – 50)  0 – 71  50 (50 – 70)  29 – 100 | 50 (50 – 50)  25 – 100  50 (50 – 50)  0 - 75 | < 0.001 |

n = Anzahl, IQR = Interquartile Range

Mögliche Abweichungen in Summen-Scores kommen durch fehlende Daten zustande.
